# 1243. Eravacycline in Bacteremia: A Case Series

**DOI:** 10.1093/ofid/ofab466.1435

**Published:** 2021-12-04

**Authors:** Justin A Andrade, Robert Kosalka, James Truong, Joshua R Rosenberg

**Affiliations:** 1 The Brooklyn Hospital Center/LIU Pharmacy, Brooklyn, New York; 2 The Brooklyn Hospital Center, Brooklyn, New York; 3 Brooklyn Hospital, Brooklyn, NY

## Abstract

**Background:**

Eravacycline (ERV) is FDA-approved for the treatment of complicated intra-abdominal infections, but there is limited experience for non-FDA approved indications.

**Methods:**

We present five cases that utilized ERV for treatment of bacteremia.

**Results:**

Patient 1 in septic shock (SS) started on vancomycin (VAN) and ceftazidime-avibactam (CZA). Blood culture (BC) finalized to *E. coli* and regimen narrowed to CZA. On day 9, gram-positive cocci in chains in BC grew and VAN was added. BC finalized to VRE *faecium* and regimen was modified to ERV on day 12. Repeat BC on day 15 finalized to no growth with no recurrence of bacteremia until discharged (day 78). Patient 2 treated for MSSA bacteremia with cefazolin and subsequent *K. pneumoniae* VAP treated with ceftriaxone (CRO) (day 18-26). On day 27, meropenem (MEM) was initiated for gram-negative bacteremia and started on IV trimethoprim/sulfamethoxazole (TMP/SMX) the following day for pneumonia caused by TMP/SMX-susceptible *S. maltophila*. BC finalized on day 29 to *S. maltophila* resistant to TMP/SMX, regimen modified to ERV. Repeat BC on day 30 finalized to no growth and ERV was continued until day 42 with no recurrence of bacteremia; however, patient died on day 45. Patient 3 with renal failure and on day 11, CRO started for SBP prophylaxis. On day 13, switched to daptomycin and cefepime (FEP) as patient was febrile and BC repeated. BC finalized to VRE *faecium* and was started on ERV on day 17 and completed a 7-day course with no recurrence of bacteremia; however, patient died on day 34. Patient 4 initially treated for bacterial superinfection with CRO and azithromycin, and subsequent worsening pneumonia treated with VAN and MEM (day 10-17). On day 19, patient was febrile and treated with VAN and FEP until day 27. Repeat BC on day 29 finalized to VRE species and modified to ERV on day 32. ERV continued for a 7-day course and was discharged with no repeat BC obtained to confirm clearance. Patient 5 in SS started on VAN and MEM. On day 3, BC on admission finalized to VRE *faecium* and therapy switched to ERV. Repeat BC taken on day 3 after ERV initiation were negative. Discharged to complete two-week course of ERV.

**Conclusion:**

ERV may be an option for bacteremia as demonstrated by clearance in four of five cases. More studies must be conducted as these reports show variable clinical outcomes.

**Disclosures:**

**Joshua R. Rosenberg, MD**, **Allergan/Abbvie** (Consultant)**La Jolla/Tetraphase** (Consultant)**Melinta** (Consultant)**Merck** (Consultant)**Paratek** (Consultant)**Sanofi** (Consultant)**Shionogi** (Consultant)

